# Antifragility and Tinkering in Biology (and in Business) Flexibility Provides an Efficient Epigenetic Way to Manage Risk

**DOI:** 10.3390/genes2040998

**Published:** 2011-11-29

**Authors:** Antoine Danchin, Philippe M. Binder, Stanislas Noria

**Affiliations:** 1 AMAbiotics SAS, CEA/Genoscope, 2 rue Gaston Crémieux, 91057 Evry Cedex, France;; 2 Natural Sciences Division, University of Hawaii, Hilo, HI 96720-4091, USA;E-Mail: pbinder@hawaii.edu; 3 Fondation Fourmentin-Guilbert, 2 avenue du Pavé Neuf, 93160 Noisy-le-Grand, France;E-Mail: stanislas.noria@gmail.com

**Keywords:** fragility, ageing, information, disorder, isoaspartate

## Abstract

The notion of antifragility, an attribute of systems that makes them thrive under variable conditions, has recently been proposed by Nassim Taleb in a business context. This idea requires the ability of such systems to ‘tinker’, *i.e.*, to creatively respond to changes in their environment. A fairly obvious example of this is natural selection-driven evolution. In this ubiquitous process, an original entity, challenged by an ever-changing environment, creates variants that evolve into novel entities. Analyzing functions that are essential during stationary-state life yield examples of entities that may be antifragile. One such example is proteins with flexible regions that can undergo functional alteration of their side residues or backbone and thus implement the tinkering that leads to antifragility. This in-built property of the cell chassis must be taken into account when considering construction of cell factories driven by engineering principles.

## 1. Introduction

The recent dread of radioactive leaks near nuclear plants reminds us vividly of the vulnerability of life. “Fragile” is an epithet often associated with living organisms, perceived as delicate and easily damaged. But does this reflect reality? What would be the opposite of fragile? In a recent piece for *Edge* magazine [1] Nassim Taleb discussed another familiar attribute of life, tinkering. There he asserted that, contrary to the favored emphasis on intelligent engineering, “*tinkering outperforms design*”. This property enables finding unexpected solutions, especially when challenged by “normal accidents” [2], rare deleterious events (in terms of individual probability of occurrence) for which he himself coined the phrase “Black Swan” events [3].

A wealth of studies compared economics and biology [4-6]. For the lay observer of economics, a point shared by both disciplines is the pervasive presence of tinkering. However, this is usually hidden behind the secrets of mathematics and computation (see an illuminating view of “Hollywood Economics” that shows how apparently unrelated individual behaviors may aggregate into coherent outcomes [7]). Tinkering is indeed at the core of life [8]. The biological evolution of unusual structures and processes in response to extreme environments or other types of challenges is ubiquitous. We explore various facets of this notion and see whether it might help us better understand the behavior and evolution of living organisms. In this context, tinkering derives from unexpected (undesigned) properties that are put in action under stressful or difficult circumstances.

Opposing fragility, properties such as “robustness” or “resilience” help make a possibly dynamic entity hard to inactivate or destroy. The expected fate of the entity is sealed: either it hardens for some time, stays the same or it breaks down. Should we extend this view to define a possibly new concept, antifragility? To define antifragility, Taleb opposes the fragility of the Damocles' Sword not to the robustness of the Phoenix (who rises from the ashes), but to the inventiveness of the Hydra (who gets two heads each time one is cut off). Is it possible to think of entities that would not just resist the ravages of time but, through the creation and recombination of novel components, actually become able to cope with an unpredictable future? More generally, can we identify processes that make an entity stronger (sturdier) through the effect of ageing, shock or stress?

In many of its features, life indeed appears to share this property with the Hydra: challenged, it responds by creating new forms of life. This property is at the root of our models of evolution. We use *dichotomies* to construct the Tree of Life. Of course, many (most) branches are cut off forever—and the Greek Hydra is not an immortal animal—but in the succession of generations, antifragile life tends to expand its exploration of the world by generating a systematic creation of novelty. This is illustrated by the huge number of new life forms that flourish following mass extinctions. On shorter time scales, we know well that it takes only a few months before anticancer drugs are defeated by cells trying to become immortal. In the same way, resistance to an antibiotic follows shortly after its introduction. Moreover, resistance does not simply result from horizontal gene transfers, using some hidden form of pre-existing resistance, but, quite often, from the emergence of a completely novel activity. For example, while the precursor of Nylon^®^ never existed before the creation of this man-made polymer, it did not take long to find bacteria that used it as a nitrogen and carbon source [9]. The enzymes involved have been created *de novo* from apparently random pieces of DNA [10]. In the same way, emerging diseases are often the progeny of pre-existing diseases. The SARS coronavirus evolved a novel surface antigen [11], and the *Escherichia coli* O104:H4 strain assembled traits that were distributed in the environment with a completely different pattern [12]. Evolution, then, should be reconsidered in this new light. Living systems are antifragile in that they can do much more than simply respond to the “pressure” of the environment by random mutations followed by selection; they have an in-built property that allows them to find solutions in the face of adversity. Antifragility is one such property. It unfolds not only in individual organisms, which age much slower than what could be expected from a fragile entity, but also in the way they generate a progeny. All these processes have in common the ability to create some novel information: antifragility cannot be separated from management of information [13].

The present reflection is organized in three parts. Rather than study multicellular organisms, where a variety of cells with complicated hierarchies display the functions of life, possibly hiding important underlying principles, we restrict our views to unicellular organisms, bacteria. We first recount the core functions, emphasizing their relative position in the process of ageing. We focus on “steady state” or “stationary” life, which we see as differing from senescence, the continuous degradation of the cell's components as time elapses (in human beings the analogous ageing-before-senescence stage would be roughly between ages 25 and 50). Subsequently we explore the process of antifragility [14] (without bothering to carry over likely challenges about the novelty of the concept) using a specific molecular example. Choosing among a variety of antifragile processes, we discuss the existence of (literally) flexible proteins and the processes in which they participate. We show how this could help delay the senescence process, providing an example of how tinkering and antifragility work together during ageing. The final discussion recapitulates the possible roles of these processes in biology at the cellular level, focusing on the stationary regime.

## 2. Functions Required to Sustain Life

The status of living organisms which do not reproduce such as the bee or ant workers stands out. Their life is limited in time, and does not perpetuate itself. In the absence of reproduction there would be no life unless it could be created spontaneously at a high rate. Were all organisms suddenly sterilized, life would continue until the last of them dies, and would then disappear. Reproduction must therefore be introduced as a core feature of what life is, beyond functions associated to stationary life. This creates a conflict between reproduction and stationary life, implying some trade-off between reproduction and survival of the individual [15]. Yet, because, by far the longest time spent by most organisms during their lifetime does not involve reproduction, we limit our exploration to life under stationary state. Indeed, if reproduction were allowed to proceed unchecked, the Earth would have been covered by one species that would have subsequently been halted in its tracks by exhaustion of nutrient supplies.

To go further, we use the Synthetic Biology engineering mode of reasoning: What would we need if we were to construct a long-lived organism? This question is essential for scaling up Synthetic Biology processes. It requires us to try and make a thorough inventory of functions, taking care not to forget unobtrusive but essential ones. To this aim, we separate cells into two independent components [16], the cell (the chassis) and its program. As reproduction is not essential during the stationary phase, we restrict our analysis to the functions of the chassis and the associated turnover of its components [17], reserving some thoughts about the constraints at the onset of reproduction to the end of this article. Note that we do not limit ourselves to structures (the term “chassis” is misleading in this respect), but explore processes as well. We further note that during the stationary phase the ever-ageing cell must either entirely freeze its metabolism and lay dormant (as a spore, for example; some Viable But Non-Culturable Cells (VBNC) might also represent this state [18]), or maintain a minimum core metabolism and energy supply, to be ready to start again multiplying when the conditions get better. The organism is in a state of flux, with specific functions involved [17]. Let us try to make a list of these functions.

### 2.1. The Cell Senses Its Physical State and Exchanges Atoms and Molecules

The cell's chassis combines management of compartmentalisation (the cell's envelope, appendages, but also its nanomachines such as the ribosome, ATP synthase, the degradosome and many others [19]) and metabolism (flux of interconversion of molecules for the building up, storage, salvage of the cell's building blocks, catalytic centers and energy management). Nutrients are imported and waste products are exported. In parallel, the cell monitors its sensors. Two types of sensing are used: sensing the physical state of the environment (temperature, light, pressure, etc) and sensing chemicals. The physical constraints cannot be neglected. Except in homeothermic organisms, changes of environmental temperature are immediately passed on to the organism. Because the effects of temperature on macromolecules are often similar to those of ageing (misfolding is the consequence of both changes), there may be considerable overlap between sensing ageing and sensing temperature variations. Indeed, molecular chaperones have both the role of sensors and of maintenance systems and they belong to the heat shock response [20]. Hence, because these molecules must act on a large number of substrates, we may assume that functions fulfilled by molecular chaperones are to be executed in priority in stationary life.

At the same time, the cell must get a large variety of chemicals from the environment. Fortunately, turnover of metabolites and salvage processes minimize the need to resort to the environment for their supply. Salvage is not a heavy-duty process. Although necessary, it should not belong to the first-line protection because few functional molecules of the corresponding enzymes are needed in the salvage process (this is akin to the marginal cost of a process in the financial world). Similarly, the time-dependent decay of most functions involved in transport of chemicals into the cell can be compensated for through considerable redundancy. The cell only needs to transport a few categories of products: amino acids, simple carbohydrates or dicarboxylates, polyamines, vitamins and ions. This is readily observed in the organisms that can live with a very low number of genes such as *Mycoplasma genitalium* [21]. Redundancy in the function of transporters, either pre-existing or resulting from the process of ageing, will limit the number needed to be conserved in the long term. Overall, in contrast with the front-line requirement for molecular chaperones, there is no heavy-duty requirement for these functions that may slowly deteriorate over time without dramatic consequences.

### 2.2. RNA is Degraded and Re-Synthesized

In contrast with the situation just described, many components have to be continuously replaced, possibly with a low turnover. This requires the continued use of the global biosynthetic capacity of the cell. Unfortunately, the identification of essential ubiquitous functions is somewhat difficult, as they cannot be solely identified using comparative genomics. While functions may be ubiquitous, enzyme structures are not [22]. In the present work we used sets of persistent genes [23] to identify the essential functions for stationary life. Consider a bacterium after it has entered stationary phase. RNAs are expected to turn over first, as they are seldom repaired (see however [24]). This process is managed by the degradosome and related activities [25]. The degradosome structure (exosome in Archaea and Eukarya) is highly diverse, being different in gamma-Proteobacteria, alpha-Proteobacteria and Firmicutes, for example [26]. It always contains polynucleotide phosphorylase (PNPase) at its core: This enzyme uses phosphorolysis rather than hydrolysis, generating nucleoside diphosphates instead of nucleoside monophosphates as basic building blocks for nucleic acids, followed by the recovery of one quantum of energy [27]. Associated to PNPase we find a variety of ribonucleases of different descent, such as RNase E in gamma-Proteobacteria or RNase J and RNase Y in Firmicutes [28], energy-dependent helicases, as well as core enzymes of the glycolysis/gluconeogenesis pathway [25]. We also find co-evolving proteins meant to degrade leftovers, such as nanoRNases of various families, depending on the phylogenetic clade [29].

A puzzling observation stems out from the composition of the degradosome: Many of its functions are carried out by proteins that are significantly longer than average bacterial proteins (300 residues). This is counter-intuitive because the synthesis of long proteins is expected to be fragile. The probability of transcription/translation errors, especially the premature termination of translation, is proportional to the length of the messenger RNA. Moreover, it is well known that truncated proteins in complexes display a negative-dominant phenotype [30]. However, the significance of this bias is supported by further observations. RNA turnover implies that the first anabolic function that must be preserved is transcription, because any requirement for re-synthesized macromolecules will first go through this step. RNA polymerase is its core component. Surprisingly, it involves two very large subunits (more than 1,300 residues), RpoB and RpoC. This might have just been a statistical coincidence, but an experiment shows that length is significant. Bacteria such as *Helicobacter pylori*, which thrive in difficult environments, fuse RpoB and RpoC into a single gigantic protein. Splitting it back into two components makes the cell sensitive to the denaturing agent urea, showing that premature termination followed by re-initiation is not an option for recovery [31]. Other important components of the machinery are also large, e.g., NusA [32] or the transcription-repair factor Mfd [33]. Is there a special property, associated with increased length that could compensate for the fragility of synthesis of long molecules?

### 2.3. Proteins also Need to be Replaced

Non-functional proteins can be either repaired [34-36] or refolded, and this costs energy. When neither is possible they are degraded. Degradation enzymes, often hydrolytic enzymes, are essential at this stage. Remarkably, while hydrolysis is a highly exothermic process, many degradation enzymes consume energy-an apparent waste. This reminds us of the remark made by Hopfield, that in order to identify important and unexpected functions, we should be exploring “*known reactions which otherwise appear to be useless or deleterious complications* [yet] *are seen to be essential to the* [studied] *function*” [37]. Antifragility implies improving while ageing, before inevitable senescence. This in turn requires a process making room to retain some sort of information.

We expect that energy-dependent degradation is the hallmark of information gathering and utilizing systems [13]. Many energy-dependent proteases have been characterized, including Clp, Lon, and FtsH (HflB) in *E. coli*. Clp proteases are degradation machines composed of a sensor required for both substrate recognition and ATP-dependent selection for unfolding, and of a peptidase made of multiple subunits, required for substrate hydrolysis (see e.g., [38]). Clp proteases (ClpP) use the ATPase subunits to choose between folded and unfolded substrates. The importance of choosing (*i.e.*, collecting information on) the substrate to be degraded is illustrated by the unexpected role of a novel class of antibiotics, acyldepsipeptides, that are active against ClpP. The acyldepsipeptide-activated ClpP core initiates proteolytic degradation without the control exerted by Clp-ATPases. This unchecked activity, which demonstrates that the energy of ATP is not used in the very process of protein hydrolysis, leads to the inhibition of bacterial cell division and eventually cell death [39]. Again, these proteins, essential during stationary life, are long.

Proteolysis makes room in the cell, and relevant feedbacks resynthesise degraded proteins that are important. To this aim, translation requires utilization of at least part of the population of ribosomes. Some ribosomes are clustered together in “hibernating” 100S complexes combining two 70S ribosomes and Rmf [40]. The available ones must have at least some functional ribosomal proteins (several are somewhat dispensable [41]). tRNA synthetases, initiation and elongation factors are also required in the process. In contrast to the proteins described previously, ribosomal proteins are short or very short proteins, a feature that could have been selected to minimize errors when they are synthesized. Why such a contrast between the translation nanomachine, and the transcription nanomachine? We shall see below that they may have features in common.

### 2.4. Even Metabolites may be Repaired

Energy is central to all the processes listed above. During stationary life the core functions of energy management (adenylate kinase and nucleoside diphosphokinase in particular) are essential to replenish the energy level of the cell; central intermediary metabolism (with synthesis of phosphoenolpyruvate and acetyl-Coenzyme A) must be present for proper functioning of the cell. Structural features of the cell's envelope must also be important. Also, the murein sacculus (the rigid peptidoglycan structure that gives the cell its shape and protects it against deleterious changes in the environment) is likely to be quite stable and will not require heavy-duty maintenance. The situation may be somewhat different for membrane phospholipids [42]. Finally, in line with the fate of macromolecules, altered metabolites are either degraded, recycled or exported to the environment. Some are repaired (AdoMet via SamT [43], D-amino acids) but this may be optional. Establishing the list of repaired metabolites should tell us something about their relative importance in the functional hierarchy of the cell under stationary conditions (Table 1).

**Table 1:** Functions, processes and metabolites that must be active during the cell's stationary phase [25].

**process**			**nanomachine**	*Escherichia coli*	*Bacillus subtilis*
maintenance					
	RNA turnover		degradosome (exosome)	Rne PnpA Eno TpiA Orn PcnB	Rny RnjA PnpA Eno TpiA NrnA NrnB
	protein turnover		proteasome	ClpAXP Lon HslVU FtsH…	ClpXP LonAB ClpCE ClpQ ClpY FtsH
	repair	refolding		Spy DnaJK GrpE GroSL…	DnaJKGrpE GroSL
		restoring		Pcm FrlDB FrlC MsrAB	FrlDB MsrAB
transcription			RNA polymerase	RpoABC NusA NusG Mfd Sigmas	RpoABC NusAG Mfd Sigmas
translation			Ribosome and tRNAs	Rps[A-U] Rpl[A-Y] Rpm[A-J] 20 tRNA synthetases Rmf EFTu EFTs EFG Modifications…	Rps[B-U] Rpl[A-Y] Rpm[A-J] 19 tRNA synthetases 1 amidotransferase EFTu EFTs EFG
		folding	chaperones	Tig Ppi DnaJKGrpEGroSL	Tig DnaJKGrpE GroSL
metabolism		carbon		Eno PykA Pps AceEF Lip Ppa …	Eno Tpi PykA PdhABC PpaC…
		nitrogen		Aminotransferases	Aminotransferases
		phosphorus		Adk Ndk Ppk…	Adk Ndk PpnKA PpnKB
compartmen-talisation		sensing transporting		Amino acids; nucleosides or bases; vitamins; carbohydrates or dicarboxylates; polyamines; ions	Amino acids; nucleosides or bases; vitamins; carbohydrates or dicarboxylates; polyamines; ions
replication	repair			chemical alterations, single and double strand breaks and recombination	chemical alterations, single and double strand breaks and recombination
	initiation		primase	control of restart	control of restart

### 2.5. Long and Short Proteins Make up the Stationary Essential Proteome, but Proteins of Average Length do not

**Figure 1:**
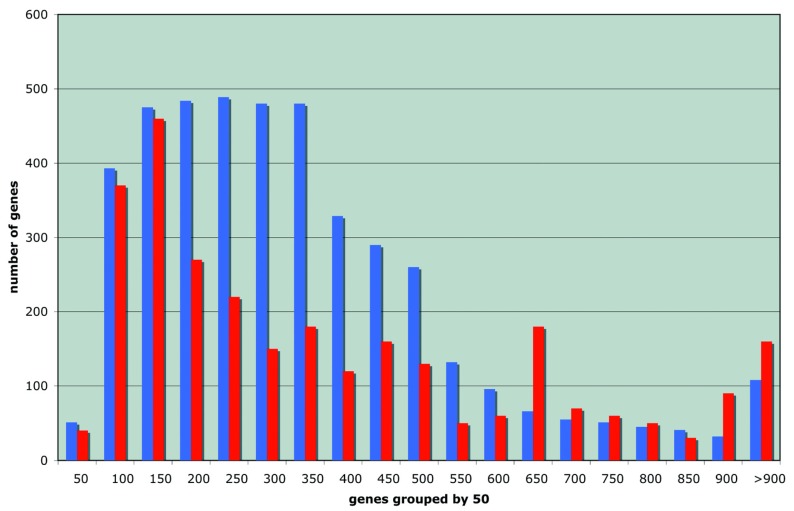
Comparative histogram of the length of the proteins of *E. coli* (in blue) and that of the functions required under stationary conditions (in red, ordinate scale multiplied by ten).

The distribution of the length of the proteins important for stationary survival is compared in Figure 1 to that of all proteins in the *E. coli* proteome: it displays a remarkable bias both for short and long proteins.

While the presence of short proteins is easy to understand (there is inherent robustness in being short as discussed by Galileo Galilei in his *Dialogo sopra i due massimi sistemi del mondo*) we need to account for the long ones. We will therefore be looking for unusual properties that could be a signature of antifragility rather than robustness. We explore now whether this unusual length distribution might suggest a direction to follow.

## 3. Processes Permitting Stress Management

Deleterious effects are often perceived as proportional to the amplitude of their causes. However, most biological systems possess some kind of buffering capacity. Provided a negative effect, a stress for example, is not too pronounced, the system reacts by preserving its homeostasis, displaying no negative change. Some effects, however, are cumulative: exceeding the buffering capacity of the system beyond a certain threshold results in accelerated weathering. In this challenging situation the cell has two choices: either its components will steadily accumulate malfunction, resulting in the error catastrophe [44], or the cell will find a solution where, provided that there are enough representatives of a given particular entity, variants will emerge, some of them ready to provide a relief to the stress induced over time.

This parallels antifragility, as proposed by Taleb to create specific detailed scenarios of financial risk management [14]. The idea behind this reflection is that when a collection of entities is exposed to serious challenges, it may be possible to obtain a positive overall outcome. Within the collection, one of the entities might fare extremely well, compensating for the collapse of some of the others and even do much better than the bulk, had it remained unchallenged. For living organisms this could act at the level of the population of organisms, the population of cells, or the population of intracellular molecules. We explore here how antifragility could operate at the molecular level, noting that its implementation has features highly reminiscent of what we call natural selection. Antifragility gathers and utilizes information [13] in the process of allowing some individual entities to stand out from the bulk and thus improving the fate of a population under a challenging situation. The number of obvious candidates for antifragile behavior among biological processes is enormous. We choose here to illustrate one of them, protein ageing, using a few specific structural features.

## 4. Flexible Molecules as Support for Antifragile Learning

Emil Fisher's lock and key model is still the paradigm of most biochemical studies. The standard view—often presented on colored images displayed on the cover of popular magazines—is that stable folding is essential not only for protein function but also for resistance to degradation. Our knowledge of proteins is significantly limited by the fact that they can be crystallized. Enzymes are perceived as rigid structures that select their substrates and synthesize products with minimal movement. The discovery of allostery changed that picture to some extent, but it still rested on the idea that activity was linked to stable and fairly rigid conformations. Yet, even crystalline proteins have “disordered” regions that do not show up in the final 3D structure, as they are invisible to X-ray diffraction due to their random distribution. Furthemore, a significant number of the proteins in the list of Table 1 are flexible or have significant flexible sections (e.g., RNA polymerase [45,46], and unexpectedly, the short ribosomal proteins [47]).

Flexibility has long been assumed in the processes of “induced fit” [48]. It reflects the selective stabilization of a conformation belonging to the spontaneous thermal fluctuations of the region of interest, leading to a final form after recruitment of proper short-range interactions (hydrogen bonds, dipolar and van der Waals interactions). Dunker and co-workers shifted the paradigm, showing that as much as one fourth of all proteins displayed a disordered structure [49]. Rather than use this negative qualifier, we here prefer “flexible”, which suggests a specific dynamic order typically relevant to processes of epistasis [50]. Despite some controversy about the role of disordered proteins, it has now been established that flexibility is part of the function of the protein, which must not interact rigidly with its targets and should be degraded once its role has been completed [51]. An example is the recent discovery of the structure and role of RNase Y in *B. subtilis* [28]. In short, function may require the selection of structures that can only appear after the fact, via proper multimolecular interactions.

### 4.1. Long Proteins have Biased Physico-Chemical Properties

Long proteins are usually multi-domain proteins connected by flexible links [52]. However, in addition to being more flexible, long proteins display remarkable structural features. Longer proteins can fulfill more easily than short ones the requirements for unfolding and misfolding stability, because they have a higher number of native interactions per residue. They are also relatively enriched in small amino acid residues. Furthermore, there is a correlation between the tendency to misfold and protein hydrophobicity, with long proteins being less hydrophobic than short ones [53]. Interestingly, when hydrophobicity is low, long proteins are more resistant to unfolding and misfolding than short proteins [54]. The collapse of unfolded polypeptides, generally believed to be driven by hydrophobic forces, is an early event in the folding of a protein. Whether hydrogen bonds and side chains play a significant role in the process has been an open question. This has recently been addressed by demonstrating that the backbone displays a considerable organizing role [55]. It seems important therefore that flexibility be a core feature of proteins involved in stationary conditions. Before discussing in more depth the way antifragility may exploit flexibility, we explore some features of flexible regions, which might be a trait selected in proteins that must solve new functional questions while ageing.

**Figure 2:**
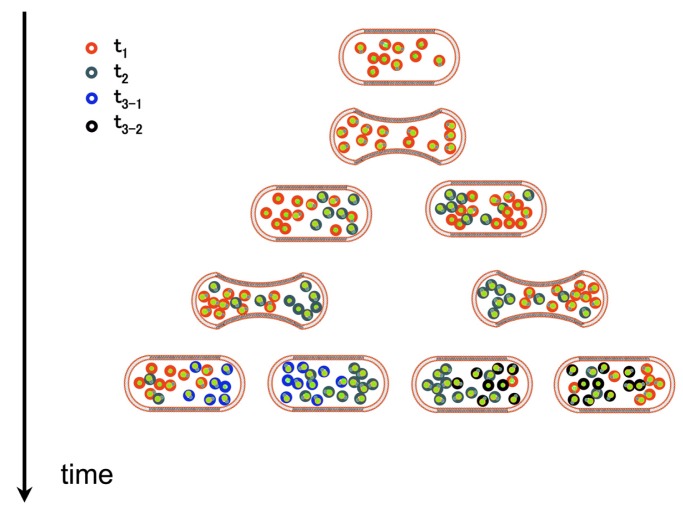
Distribution of proteins of different ages as cells multiply. At the onset of growth the cell is supposed to have all of its proteins of a given type of identical age (red circles). As time elapses some proteins begin to age (green, then blue and purple circles) and are replaced by young ones (red circles). At some point all cells display a mixture of the same protein carrying scars marking their different ages.

Birth, growth, maturation and senescence are the four ages of all cells. This is true of their components as well. In general the maturation step is ignored: cell's components are viewed as synthesized, used in their final form, then decaying and being either repaired or destroyed. Maturation and possibly functional improvement during ageing is rarely taken into account. Senescence and ageing are treated as equivalent. Yet, quite a few physico-chemical processes suggests that the state of cell components at any time should be seen as actively browsing through a series of ageing states. In fact, notwithstanding apoptosis (which may be a process to reset the system), most cells harbor a mixture of aged and young components, reflecting the overall history of their divisions (Figure 2).

Flexibility plays a considerable role here. We explore the notion that, before the deleterious senescence process is triggered, a process of maturation will create a population of molecules that will undergo different fates. Flexible regions in some cases will make the protein prone to be degraded by proteolytic systems. In other cases, via interaction with specific partners, it will distribute the protein among functional entities. Finally, facing a variety of challenges, flexibility might uncover novel properties of the protein that need time to be implemented. Hence the different individuals in a collection of a given gene product may have different fates, and react differently to similar challenges.

### 4.2. The Function of Post-Translational Modifications is Disputed

Two processes are generally considered important in the maturation of macromolecules: metabolism-driven modifications and intrinsic instabilities. Post-transcriptional and post-translational modifications show that some metabolism-dependent changes are genetically programmed. At the same time, basal metabolism is required to allow the cell to maintain its activity. Many metabolites are not innocuous. Some have intrinsic reactivity: dicarbonyls (including reducing carbohydrates) react with accessible amino groups resulting in glycation [56] and respiration produces free radicals or reactive oxygen species (ROS) that modify nucleic acids and proteins. The role of ROS is often perceived as negative. However, in addition to being a potential threat to cellular integrity, ROS are also used in a programmed way by the cell, for example in insects, where ROS-triggered protein alteration is required for the formation of the exoskeleton [57]. In the same way, laccase is involved in formation of the spore coat defense in *Bacillus subtilis* [58]. Superoxide is essential for longevity in *Caenorhabditis elegans* [59] and there is evidence in mice that several oncogenes actively promote a ROS detoxification program that is required for tumour initiation [60]. This raises questions such as: Is the ROS-dependent modification of a common amino acid, such as methionine oxidation, programmed or accidental [61,62]? Is an oxidized protein a prelude to senescence or a form of the protein that has evolved specifically with the function of regulation, for example?

Unprogrammed modification of macromolecules is often seen as leading to loss of function. This is certainly true of most modifications caused by reactive metabolites such as free radicals, but many modifications are programmed and used as control processes (phosphorylation in particular) or protection processes (methylations or acetylations [63]). Some, such as lysine carbamylation [64] or hypusinylation [65], are even essential for activity. Self-cleavage and formation of a pyruvoyl active center exists in proteins present in the three domains of life [66], inteins are spliced out of ribonucleoside diphosphate reductases [67], reactive centers are made of covalent adducts [68,69]… One also observes more subtle changes in the backbone of macromolecules such as (deoxy)ribose puckering in nucleic acids [70,71], proline puckering [72], isomerisation of aspartate and asparagine with associated deamidation [73], and there is considerable evidence that this could be functional.

Interestingly, the cell's components weather with variable rates (possibly programmed in the very sequence of informational biopolymers [74]), depending on their chemical nature and on their flexibility. This process occurs with a definite half-time, specific of the protein sequence. It allows the protein to display the possibility for time-measurement, *i.e.*, behave as a clock coupled to a structural change. This allows the protein to change its interaction with relevant control mediators, at a pre-set time. Clocks are central devices in information processing. In particular they allow parallel processes the opportunity to synchronize. Accurate timing requires the presence of a stable clock-an unlikely feature of living cells-but cooperation of many inaccurate clocks may result in defining a proper timing [75], possibly to tell cells that they have to start expressing new regulatory pathways or initiate replication. Because this onset of a variety of behaviors which happens after the protein has been synthesized, under conditions when challenges may be taken into account, this process may promote antifragility.

Long proteins such as RNA polymerase subunits have flexible regions and their structure (and sequence) could then change as time elapses. Their length gives them features that make them robust [54], but could they improve over time? Should not we reinvestigate their structure/function in ageing cells? In the same way, repair systems are made of long proteins, and these proteins need to avoid the damages of age because they are essential to prevent premature death [76]. Rather than the cause of modification-derived senescence, it seems worth investigating whether flexibility and length could be the hallmark of antifragility. Indeed, for example, large unstructured hydrophilic flexible regions or proteins provide cellular resistance to dehydration [77].

### 4.3. Isomerisation of Asparagine and Aspartate Residues Creates Context-Dependent Clocks

**Figure 3:**
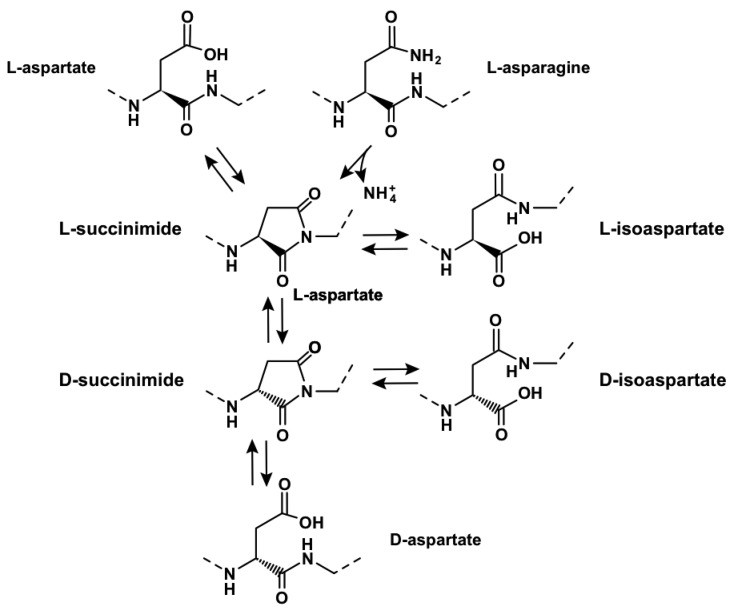
Ageing of aspartate and asparagine residues in the protein backbone.

To illustrate concretely these points, here is a detailed example of a specific time-dependent post- translational molecular mechanism. Flexibility favors cyclisation of aspartate and asparagine residues into L-succinimide (Figure 3).

This process is both fast and frequent, in particular at asparagine-glycine (AsnGly) motifs because of the intrinsic flexibility of glycine [73], with an associated deamidation of asparagine. L-succinimide will either hydrolyse into L-isoaspartate spontaneously or after methylation (in 2/3 of the cases), or L-aspartate (in 1/3 of the cases). Subsequently, L-succinimide will isomerise into D-succinimide at a slow rate, and then lead to formation of D-isoaspartate and D-aspartate residues. As a consequence, proteins with flexible regions containing aspartate or asparagine residues will considerably change over time, leading to multiple states, depending on the past history of the protein. Remarkably, the beta subunit of RNA polymerase contains conserved AsnGly motifs. Because of the cost of resynthesising the protein, if destined to become rapidly non-functional after cyclisation-deamidation, these motifs should have been genetically rejected rather than preferred, unless deamidation is of positive biological value. In the same way, aspartates modified by phosphorylation or methylation are also prone to cyclisation. This may lead to loss of activity, creation of novel functional properties, such as regulatory properties or novel catalytic features (reviewed in [73]).

Many other changes occur as time elapses, in particular during the catalytic cycles in which enzymes perform their functions. In some cases, once a protein has been modified (e.g., alkylation of a cysteine residue in the Ada enzyme dealkylating DNA [78]), it must be degraded and resynthesized. However, this is extremely costly and should be limited to some rare situations when it is better to lose energy than control. This cannot be the rule, and inevitable time-dependent changes in macromolecules provide good reasons to suspect that some have been recruited for being functional in the long run, and even to alter their function in a context-related positive direction, allowing them to display antifragile features. This could account for the bias towards long proteins among those that are needed during stationary ageing, the maturation process preceding deleterious senescence.

## 5. Conclusions

Drawing an image of the environment is a way to foresee the future. This is illustrated in the way living organisms readily cope with changes in their environment, anticipating what it should be like, measuring differences with expectations and adjusting to reality. We propose that antifragility provides a way to allow the viability of cells and organisms in the long run by providing flexibility in structures and processes so that said cells can learn and implement versatile solutions to the problems posed by a variable environment during stationary life.

Extending the concept of induced-fit, we can see antifragile flexible structures and processes as molded on particular ordered structures and processes of the organism. Antifragility could use pre-set properties selected during evolution, such as the retention of asparagine or aspartate residues at specific positions in proteins, and a context-dependent ability to cyclize. This way, the cell could learn and memorize a particular moment in its history, and fixate it so that it could be recalled later on when similar conditions appear. Antifragility would thus be a way to implement a form of epigenetic memory and contribute to the overall mechanisms of network epistasis [50]. In the present context it has properties similar to those used in the market of financial derivatives: the cell develops a diverse population of molecules of processes such that, under challenging conditions, one or more will emerge and improve the cell's fate in a much better way than a uniform population would have [14]. At this point, an aged cell, when facing suddenly a medium where it could reproduce, would initiate a round of replication. We did not discuss here the corresponding required processes and functions, which may underlie the widely spread observation of VBNC [18], but the present view should be seen as a pre-requisite to explore this stage of life that represents up to 99% of the bacteria present in the environment.

This population view, with special properties coming from a few individuals, asks for a way to extract a common underlying order that can be memorized. How could order be identified? Remarkably, there exists a built-in property of large systems of relationships that may answer the question. The cell is constantly in the midst of a huge network of interacting partners. This constitutes an evolving graph. Can we identify some order in the graph? An important feature of large interaction graphs is that they comply with Ramsey's theory, which states that a sufficiently large system, no matter how random, must contain highly organized subsystems [79]. Antifragility is context- dependent. But there is no means for an antifragile structure to know whether the constraints that made it evolve came from the outside or from the inside. Antifragile structures and processes that would change state when some order is reached could memorize a diagram of that particular order. In this way, once time has taken its toll on the organism, a network of interactions would have been created via antifragile modifications of multiple elements, allowing it to trigger re-synthesis of some components that would fit in place in the graph, because the interaction graph itself would play the role of the original situation that enabled its creation.

This behavior is similar to that of the brain, which both records immediate cues in the environment and anticipates its future behavior without knowing whether the stimuli are endogenous or exogenous. This is performed via a set of hierarchical functions that combine bottom-up recording (from the senses to the higher levels of the brain) with top-down feedback used to prepare the sensing apparatus to what might soon appear [80]. An antifragile entity will sense and record “on the fly” memories from the bottom up (for example asparagine cyclisation and deamidation will depend on temperature changes, frequency of the involvement of catalytic activity). And gene expression will adjust, top- down, the answer of the cell as a whole.

When accounting for an immediate reaction to challenge, antifragility thus contributes to fitness. A remarkable property of antifragility is the fact that, by definition, an antifragile entity will react to changes by providing a solution that is not exactly programmed. Hence it does not have a fixed fitness: its response is context and time-dependent. This type of behavior is also the hallmark of tinkering, giving credence to Taleb's reflection.
